# Is There Any Difference in Preferences Between Patients and Physicians? Evidence From Anti‐Hyperglycemic Medications Choices for Type 2 Diabetes

**DOI:** 10.1111/1753-0407.70220

**Published:** 2026-04-15

**Authors:** Yuliang Xiang, Liu Liu, Jing Liu, Xiong Ke, Yanfeng Ren, Shiyi Bao, Fuming Li, Shimeng Liu, Yingyao Chen

**Affiliations:** ^1^ School of Public Health, Fudan University Shanghai China; ^2^ National Health Commission Key Laboratory of Health Technology Assessment Fudan University Shanghai China; ^3^ Fudan Institute of Belt and Road & Global Governance, Fudan University Shanghai China; ^4^ School of Management, Hainan Medical University Haikou Hainan China; ^5^ School of Management, North Sichuan Medical College Nanchong Sichuan China; ^6^ Jincheng Center for Disease Control and Prevention Shanxi China; ^7^ Department of Teaching Management Nanjing Medical University Jiangsu China

**Keywords:** anti‐hyperglycemic medications, discrete choice experiment, patient preferences, physician preferences, shared decision‐making

## Abstract

**Aims:**

This study aimed to explore differences in preferences between Chinese patients with type 2 diabetes mellitus (T2D) and physicians when selecting anti‐hyperglycemic medications.

**Materials and Methods:**

We conducted a discrete choice experiment (DCE) involving 1784 patients (face‐to‐face surveys) and 168 physicians (online questionnaires) across eastern, central, and western China. Seven treatment attributes were assessed: HbA1c reduction, hypoglycemia risk, cardiovascular benefits, gastrointestinal adverse events, weight change, mode of administration, and monthly out‐of‐pocket cost. Preference heterogeneity was explored using latent class model.

**Results:**

Patients placed the greatest importance on monthly out‐of‐pocket cost, treatment efficacy, and hypoglycemia risk, whereas physicians prioritized hypoglycemia risk, cardiovascular benefits, and treatment efficacy. Notably, physicians tended to overestimate patients' willingness to pay for treatment benefits. Specifically, patients were willing to pay 277 CNY (39 USD) for cardiovascular benefits, whereas physicians estimated that patients would be willing to pay 864 CNY (121 USD). Latent class analysis identified substantial heterogeneity in both groups. In particular, the largest patient subgroup was strongly cost‐sensitive, with preferences driven primarily by financial burden, whereas no directly corresponding physician subgroup was observed.

**Conclusion:**

Patients and physicians differ in how they prioritize diabetes medication attributes, especially regarding efficacy, cardiovascular benefit, and cost. Shared decision‐making should account for these differences to support value‐concordant, patient‐centered diabetes care.

## Highlights

This study reveals substantial differences in anti‐hyperglycemic medication preferences between Chinese patients with T2D and physicians. Patients prioritized out‐of‐pocket cost and treatment efficacy, while physicians emphasized hypoglycemia risk and cardiovascular benefits. These discrepancies highlight the need for enhanced shared decision‐making to align treatment choices with patient values and improve adherence.

## Graphical abstract

Differences in patient and physician preferences for anti‐hyperglycemic medication attributes in China.

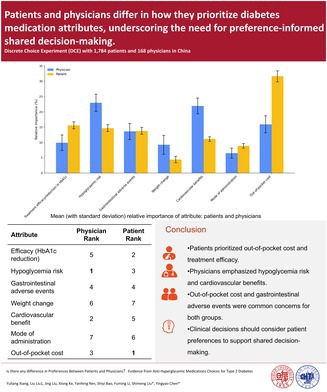

## 1 Background

Over recent decades, clinical management has increasingly shifted toward patient‐centered care, with an emphasis on shared decision‐making [1, 2]. As part of this process, there has been a growing recognition of the importance of understanding patient preferences in treatment selection. This approach is particularly relevant for diseases with multiple feasible treatment options where no single therapy has proven superior, Type 2 diabetes mellitus (T2D) is one such condition [3, 4, 5, 6].

Metformin is widely recommended as the first‐line therapy for T2D [7]. However, due to the progressive nature of the disease, approximately 50% of patients require additional anti‐hyperglycemic medications within 3 years of initiating monotherapy [8]. Several second‐line medications differ in efficacy, side effect profiles, administration routes, and costs [9, 10]. Importantly, beyond patients with established atherosclerotic cardiovascular disease (ASCVD), high ASCVD risk, heart failure, or chronic kidney disease—where guidelines recommend SGLT2 inhibitors or GLP‐1 receptor agonists for cardiovascular benefit—clinical guidelines provide flexibility rather than definitive recommendations for selecting second‐line agents in other patient populations [7, 11]. In this study, “second‐line therapy selection” refers to the common decision point of initiating an additional glucose‐lowering agent after first‐line therapy (typically metformin) when glycemic targets are not met and no overriding cardiorenal indication mandates a specific drug class. This highlights the critical role of incorporating patient preferences into treatment decisions at this stage. The American Diabetes Association also emphasizes that the choice of add‐on therapy should be guided by both clinical characteristics and patient preferences [7].

T2D presents unique challenges in achieving treatment decisions that align with patient preferences. As a chronic disease requiring long‐term self‐management, patients' concerns about weight gain, hypoglycemia, treatment burden, and quality of life may differ from physicians' priorities [12], which often emphasize long‐term clinical outcomes and adherence to evidence‐based guidelines [13]. Understanding and reconciling these differences in preference is essential for achieving truly patient‐centered care [14, 15]. Evidence suggests that alignment between patients and physicians' preferences can enhance treatment adherence and satisfaction, ultimately contributing to improved health outcomes [16, 17]. Previous studies have primarily focused on patients' preferences regarding treatment choices for T2D [4, 5, 6, 12, 18, 19, 20, 21, 22, 23]. However, the differences in preferences between patients and physicians have not been thoroughly investigated.

The present study conducted a discrete choice experiment (DCE) to quantify medication preferences of patients and physicians in the selection of second‐line anti‐hyperglycemic medications. This is the first DCE study in T2D specifically designed to explore differences in preferences between patients and physicians. Findings from this study may contribute to the shared decision‐making process and support the development of patient‐centered decision aid for patients with T2D.

## 2 Methods

### 2.1 Study Design

In this survey study, two comparable questionnaires were developed to assess the medication preferences of patients and physicians. Patient data were collected via face‐to‐face surveys conducted at healthcare institutions, whereas physician data were collected online through the Sojump survey platform. Completion of the questionnaires took approximately 10–15 min for physicians and 15–20 min for patients. All responses were collected anonymously. The ethics approval was obtained from the institutional review board of a leading academic institution in China (Reference No. IRB# 2021‐07‐0911), and the research adhered to the tenets of the Declaration of Helsinki. All patients provided their informed consent prior to their inclusion in the study.

### 2.2 Study Sample

Following the widely used Johnson and Orme rule‐of‐thumb for discrete choice experiments, the minimum required sample size can be approximated as $N\geq 500\times c/\left(t\times a\right)$, where *c* is the largest number of levels for any attribute, *t* is the number of choice tasks per respondent, and *a* is the number of alternatives per choice task. For patients, we set *c* = 6, *t* = 6 choice tasks (excluding one repeated choice set for internal validity), and *a* = 2, resulting in a minimum required sample size of approximately 250. For physicians, who completed 12 choice tasks with the same attribute structure (*c* = 6, *a* = 2), the corresponding minimum required sample size was approximately 125. The realized sample sizes for both groups exceeded these minima. Considering regional variations in diabetes prevalence and economic development levels across China, we employed a multistage stratified cluster sampling method to collect patient data. First, three provinces representing Eastern, Central, and Western China were selected. Second, from each province, one provincial capital city (economically developed) and one non‐capital city (economically underdeveloped) were chosen. Third, one or two hospitals and two or three primary care institutions were randomly selected from each city, totaling 28 healthcare institutions from six cities. Patient participants were eligible to complete the survey if they were Chinese, ≥ 18 years of age and diagnosed with T2D by a healthcare professional. Individuals were asked to participate regardless of their medication history.

Physician data were collected through an online questionnaire distributed via professional medical associations and healthcare platforms. An online survey was selected to leverage the high education level and digital literacy of the physician population, and to enhance efficiency and geographic reach of data collection [24]. Physicians were recruited from the same provinces and regions where patient data were collected to ensure consistency and comparability between the two groups. To be eligible, physicians had to be 18 years of age or older, hold a license to practice medicine in China, specialize in endocrinology or general practice, and have managed at least one patient with T2D in the preceding year.

### 2.3 Discrete Choice Experiment

In this study, we adhered to recommended good research practices for health‐related DCEs, including clear definition of the decision context, systematic development of attributes and levels, piloting of the questionnaire, and use of internal validity tests [25]. Patient and physician preferences of anti‐hyperglycemic medications for T2D were assessed using a discrete choice experiment (DCE). In a DCE, participants complete a series of choice tasks where they select their preferred option from hypothetical treatment alternatives varying systematically in attributes and levels relevant to the choice of anti‐hyperglycemic medications. The selection of attributes for patients was informed by our previous review of the published literature and best‐worst scaling (BWS) study involving 362 patients with T2D [21]. This study quantitatively ranked attribute importance from the patient perspective. These attributes were further refined through focus‐group discussions with six endocrinologists. To facilitate direct comparison between patient and physician preferences, based on the literature review, expert consultation, BWS results and focus‐group interview, the same set of seven attributes was used in the discrete choice experiment (DCE) for both groups: treatment efficacy, weight change, hypoglycemic risk, gastrointestinal adverse events, cardiovascular benefits, mode of administration, and monthly out‐of‐pocket cost (Table 1). Attribute levels were defined according to clinical evidence, guidelines, and local health insurance data, consistent with our previously established methodology.

**TABLE 1:** Attributes and levels in the discrete‐choice experiment survey.

Attributes	Levels	Description	Some corresponding medications
Treatment efficacy/reduction in HbA1c	Highest/2.5%	Different diabetes drugs have different efficacies for reducing the HbA1c	Insulin
	High/1.5%		Insulin, GLP‐1 RA, SGLT‐2i
	Intermediate/1.0%		Sulfonylureas, nateglinide, biguanides
	Poor/0.5%		TZD, alpha‐glucosidase inhibitor, DPP‐4i
Hypoglycemic risk	0%	The likelihood that patients will experience mild or moderate hypoglycemic events within the first 6 months of use	TZD, alpha‐glucosidase inhibitor
	5%		Nateglinide
	15%		Sulfonylureas
	30%		Insulin
Gastrointestinal adverse events	0%	The likelihood that the medication will cause any mild or moderate GI adverse events (which may include diarrhea, vomiting and/or nausea) within the first 6 months of use.	DPP‐4i, SGLT‐2i
	10%		Meglitinide
	20%		Alpha‐glucosidase inhibitor
	40%		GLP‐1 RA
Cardiovascular benefits	Yes		GLP‐1 RA, SGLT‐2i
	No		Others
Weight change	−2 kg	Medication‐related weight changes that patients will experience within the first 6 months of use.	SGLT‐2i, GLP‐1 RA
	0 kg		DPP‐4i, alpha‐glucosidase inhibitor
	+1.5 kg		Sulfonylureas, nateglinide, TZD
	+3 kg		Insulin
Mode of administration	Injection	—	Insulin, most GLP‐1 RA
	Pill		Others
Out‐of‐pocket cost#	¥0	Patients' monthly out‐of‐pocket cost.	Sulfonylureas, glucosidase
	¥50		TZD, nateglinide
	¥100		Alpha‐glucosidase inhibitor
	¥200		DPP‐4i, SGLT‐2i
	¥400		Most insulin
	¥600		Some insulin, GLP‐1 RA

*Note:* #Based on a currency exchange rate of 7.12 CNY to 1.00 USD in 2024.

Both patient and physician questionnaires included a dominance (quality control) choice task with one clearly superior option; respondents who failed this task were excluded from the final analysis. To ensure adequate understanding of technical terms, all patient interviews were administered face to face by trained interviewers. For the online physician survey, we additionally applied a minimum completion‐time threshold to screen out implausibly fast responses.

### 2.4 Experimental Design and Design of DCE Choice Tasks

The DCE experimental design was generated using a D‐optimal algorithm, resulting in a total of 48 choice tasks. These tasks were divided into 8 blocks, each containing 6 choice tasks. The primary goal of this design was to ensure that each participant responded to a sufficient number of questions for robust parameter estimation, whereas minimizing respondent burden. Each participant was randomly assigned to one block consisting of 6 choice tasks, with each task requiring participants to select one preferred option from two hypothetical medication profiles.

### 2.5 Statistical Analysis

Statistical analysis of the DCE data employed conditional logit models (CLM) and mixed logit models (MIXL). The best‐fitting model was selected based on Bayesian Information Criterion (BIC) and Akaike Information Criterion (AIC). Marginal willingness‐to‐pay (mWTP) was calculated, treating the cost attribute as a fixed and continuous variable. For physicians, mWTP reflects the amount they believe is reasonable for patients to pay, rather than their own out‐of‐pocket expenditure. Preference heterogeneity among participants was assessed using latent class analysis (LCA). LCA identified distinct subgroups of participants with varying preferences for medication attributes, determined the optimal number of classes, and estimated class‐specific preference weights. All statistical analyses were conducted using Stata 17.0 software.

## 3 Results

A total of 1793 patients participated in the DCE, of whom 1784 completed all assigned choice tasks and were included in the final analytic sample. The median age of patients was 61 years, and 49.1% were female. Regarding diabetes duration, 16.6% had been diagnosed for less than 3 years, 20.2% for 3–5 years, 26.7% for 5–10 years, and 36.5% for more than 10 years. More than 75% of patients reported monthly out‐of‐pocket costs for anti‐hyperglycemic medications ranging from ¥50 to ¥600.

A total of 208 physicians participated in the DCE, of whom 168 completed all assigned choice tasks and were included in the final analytic sample. The median age of physicians was 38 years, and 54.8% were female. Overall, 38.7% of physicians reported having more than 10 years of experience in endocrinology.

The AIC and BIC values suggested that the MIXL outperformed the CLM. All treatment attributes levels significantly influenced treatment preferences in both patients and physicians (*p* < 0.05; Table 2). Among physicians, greater HbA1c reduction, lower side effect risks, and cardiovascular benefits had significant positive impacts on treatment preference. The highest preference was for 0% hypoglycemia risk (*β* = 0.98, *p* < 0.01), followed by cardiovascular benefit (*β* = 0.94, *p* < 0.01) and a 2.5% HbA1c reduction (*β* = 0.35, *p* < 0.01). Physicians also preferred oral administration (*β* = 0.28, *p* < 0.01), whereas weight change had no significant effect. Increased cost reduced preference (*β* = −0.11, per 100 CNY, *p* < 0.01). Among patients, preference was most influenced by 0% hypoglycemia risk (*β* = 1.11, *p* < 0.01), followed by cardiovascular benefit (*β* = 0.84, *p* < 0.01) and a 2.5% HbA1c reduction (*β* = 1.18, *p* < 0.01). Patients preferred oral medications (*β* = 0.67, *p* < 0.01) and showed a negative preference for weight gain, particularly at +3 kg (*β* = −0.34, *p* < 0.01), whereas preference for weight loss was not significant. Higher cost decreased preference (*β* = −0.40, per 100 CNY, *p* < 0.01). Physicians had a more positive preference toward reducing treatment risk than improving efficacy (0.98 vs. 0.42), whereas patients displayed a less positive preference toward reducing treatment risks than improving efficacy (1.11 vs. 1.18).

**TABLE 2:** Attribute‐level preference weights.

Attributes	Levels	Physicians		Patients	
		Estimate ± SE	SD	Estimate ± SE	SD
Treatment efficacy/reduction in HbA1c	0.5% (ref.)				
	1%	0.29 ± 0.13*	0.3	0.69 ± 0.07**	0.84**
	1.50%	0.42 ± 0.11**	0.05	1.08 ± 0.09**	0.86**
	2.50%	0.35 ± 0.13**	0.53**	1.18 ± 0.09**	0.64**
Hypoglycemic risk	30% (ref.)				
	15%	0.47 ± 0.12**	0.52*	0.65 ± 0.07**	0
	5%	0.61 ± 0.12**	0.02	0.88 ± 0.07**	0.46*
	0%	0.98 ± 0.12**	0.38	1.11 ± 0.09**	0.35
Gastrointestinal adverse events	40% (ref.)				
	20%	0.11 ± 0.12	0.06	0.34 ± 0.06**	0.12
	10%	0.38 ± 0.12**	0.13	0.57 ± 0.08**	0.86**
	0%	0.58 ± 0.11**	−0.06	1.04 ± 0.09**	1.13**
Weight change	No change (ref.)				
	−2 kg	0.09 ± 0.11	0.25	−0.03 ± 0.07	0.34
	+1.5 kg	−0.40 ± 0.13**	0.50*	−0.22 ± 0.07**	0.51**
	+3 kg	−0.20 ± 0.11	0.08	−0.34 ± 0.08**	1.07**
Cardiovascular benefits	No (ref.) Yes	0.94 ± 0.11**	0.95**	0.84 ± 0.06**	0.71**
Mode of administration	Injection (ref.) Pill	0.28 ± 0.07**	0.32*	0.67 ± 0.06**	1.25**
Out‐of‐pocket cost#	Increase 100 CNY	−0.11 ± 0.04**		−0.4 ± 0.04**	

*Note:* *p ≤ 0.05; **p ≤ 0.01; #Based on a currency exchange rate of 7.1217 CNY to 1.00 USD in 2024.

The mWTP analysis quantified the monetary amount that patients were willing to pay, or that physicians thought patients should pay for the desired level of an anti‐hyperglycemic medication attribute compared with the reference level. mWTP estimates are presented in Figure 2.

**FIGURE 1:**
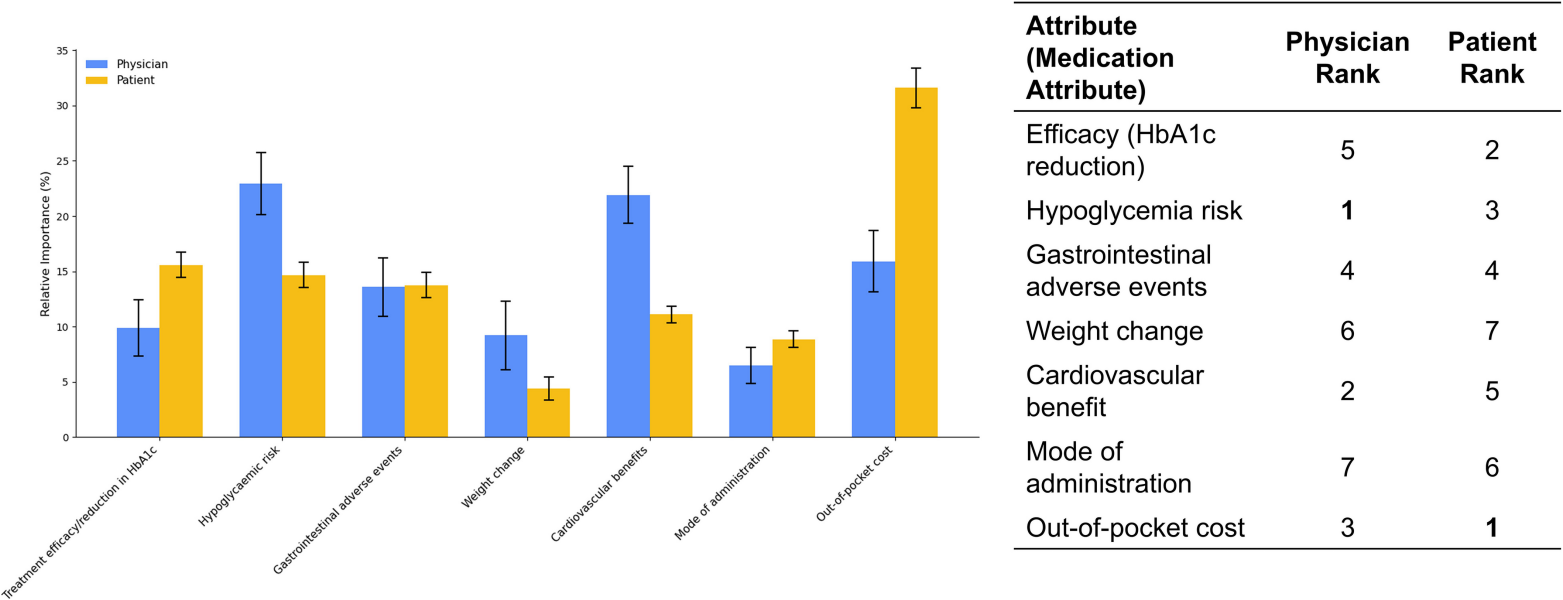
Mean (with 95% CL) relative importance of attribute: Patients and physicians.

Figure 1 shows the mean relative importance of attributes for patients and physicians. Patients placed the highest importance on out‐of‐pocket cost (31.6%), followed by treatment efficacy in terms of HbA1c reduction (15.6%), and hypoglycemic risk (14.7%). In contrast, physicians ranked hypoglycemic risk as the most important attribute (22.9%), followed by cardiovascular benefits (21.9%), and treatment efficacy (9.9%).

**FIGURE 2:**
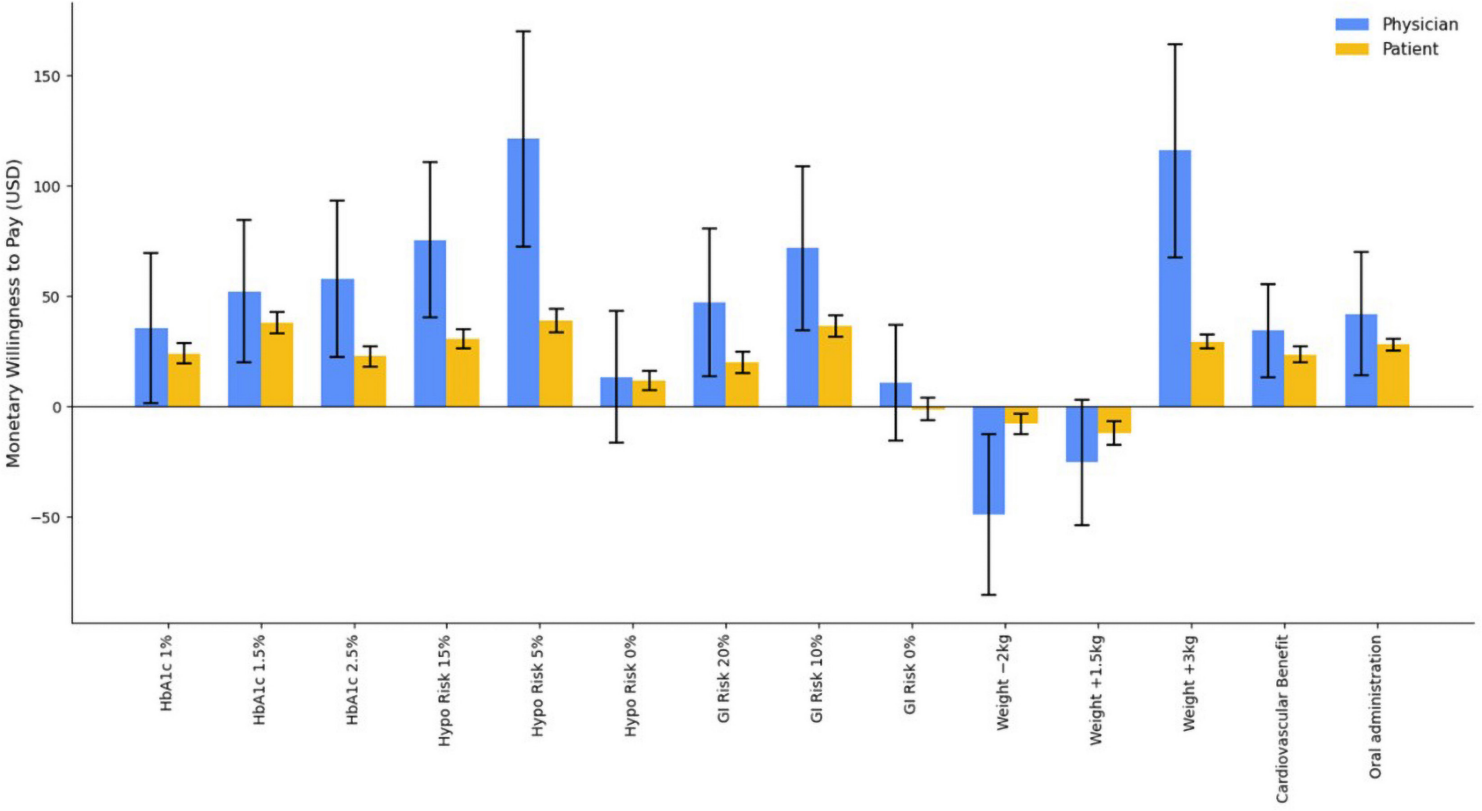
Marginal willingness to pay. Based on a currency exchange rate of 7.12 CNY to 1.00 USD in 2024.

**FIGURE 3:**
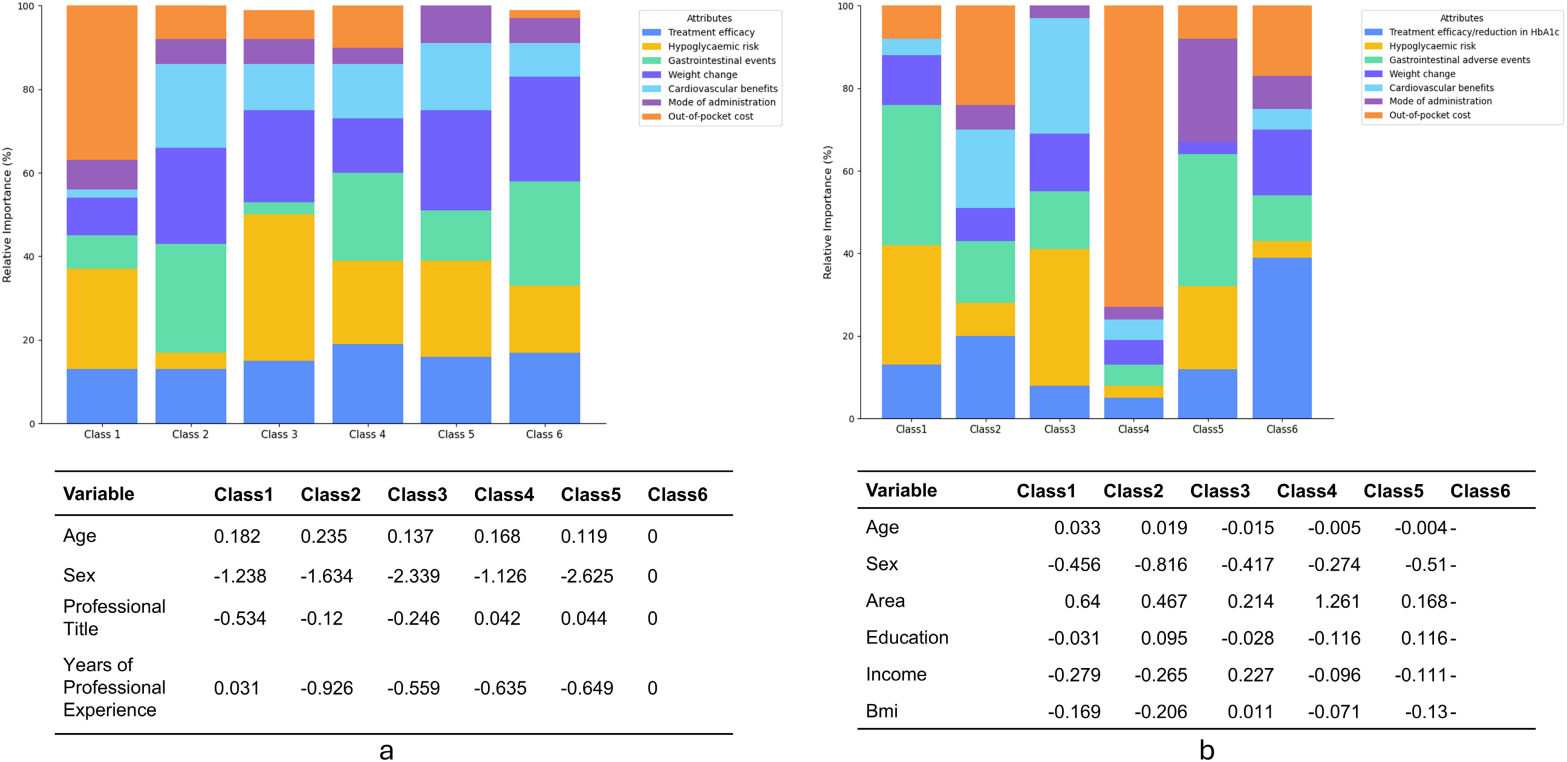
Latent class model for preferences heterogeneity. (a) latent class model for physicians; (b) latent class model for patients.

Latent class analysis identified six distinct classes of physicians, with class membership probabilities of 13%, 21%, 15%, 17%, 11%, and 23% for classes 1 through 6, respectively (Figure 3; Table S4, Table S5). Physicians in Class 1 placed the highest importance on out‐of‐pocket cost and were influenced by hypoglycemia risk, gastrointestinal adverse events, and cost‐related concerns. Class 2 physicians were less sensitive to hypoglycemia risk and prioritized gastrointestinal adverse events, weight change, and cardiovascular benefits. Class 3 focused strongly on reducing hypoglycemia risk and preferred oral administration. Class 4 physicians showed overall low levels of preference across attributes, suggesting less differentiated decision‐making. Class 5 emphasized avoidance of cost and adverse effects, including gastrointestinal and hypoglycemia risks. Class 6 showed the strongest preference for treatment efficacy, along with hypoglycemia and gastrointestinal risk, and weight change. A class membership model was conducted using Class 6 as the reference. Compared to Class 6, male physicians were significantly more likely to belong to any of the other classes, with sex coefficients ranging from −1.1 to −2.6. No consistent associations were observed for professional title or years of experience.

Latent class analysis also revealed six patient classes with class probabilities of 16%, 16%, 13%, 31%, 11%, and 14%, respectively. Patients in Class 1 showed low concern for mode of administration and were mainly influenced by hypoglycemia and gastrointestinal risks. Class 2 placed the greatest emphasis on out‐of‐pocket cost, with additional consideration given to treatment efficacy and cardiovascular benefits. Class 3 emphasized both hypoglycemia risk and cardiovascular benefits. Class 4 (the largest group) exhibited the strongest aversion to cost, with preferences primarily driven by financial burden. Class 5 prioritized avoidance of gastrointestinal adverse events, along with concerns about hypoglycemia and mode of administration. Class 6 focused predominantly on treatment efficacy.

In the class membership model (reference: Class 6), female patients were more likely to belong to Class 1, 2, 3, and 5. Higher income was associated with Class 3 membership. Notably, patients residing in rural areas were more likely to belong to Class 1 and Class 4 (*β* = 0.64 and 1.26, respectively), indicating greater cost sensitivity and potentially different care expectations compared with urban patients.

## 4 Discussion

This study utilized a DCE to investigate preferences for anti‐hyperglycemic medication attributes among Chinese patients with T2D and physicians. Consistent with the random utility framework, all attributes contributed meaningfully to respondents' choices. However, notable differences emerged regarding the prioritization of these attributes between patients and physicians. Beyond confirming that patients value efficacy, hypoglycemia risk, and out‐of‐pocket costs, consistent with previous DCEs in Chinese T2D patients [22, 26, 27], this study extends the literature in two important ways. First, by administering an identical attribute framework to both patients and physicians, it provides a direct quantification of preference divergence between the two groups, rather than inferring differences from separate studies. Second, by combining DCE with latent class analysis, it uncovers distinct preference phenotypes within each group that are not apparent from average estimates alone, highlighting the need for more tailored shared decision‐making strategies.

Previous studies have consistently shown that physicians and patients often differ in how they value treatment outcomes and risks, with physicians frequently misjudging what matters most to patients [15, 28]. The pattern observed in our study—patients attaching more importance to treatment efficacy and out‐of‐pocket cost, and physicians emphasizing hypoglycemia risk and cardiovascular benefits—is consistent with this broader misalignment. Several mechanisms may underlie this discordance. First, information asymmetry and different risk perceptions may lead patients to focus on immediate affordability and short‐term glycemic control, whereas physicians may overweight low‐frequency but high‐consequence harms under professional accountability and guideline‐driven practice. Second, prescribing is shaped by institutional and payer constraints (e.g., formulary access and reimbursement rules), which can shift physicians' attention toward safety and long‐term outcomes even when patients' day‐to‐day budget constraints dominate their choices. These findings suggest that diabetes care should not be organized around a single “average” patient or a uniform set of value assumptions. Preference data such as ours can complement clinical evidence when developing care pathways and quality indicators, and can motivate the use of structured shared decision‐making at the consultation level so that individual treatment choices better reflect the diversity of patient values.

In addition, patient education may strengthen preference‐based shared decision‐making [29]. Our findings reveal heterogeneity in treatment preferences, with some patients prioritizing immediate attributes such as cost and short‐term glycemic control over long‐term benefits including cardiovascular protection and hypoglycemia prevention. Although these patterns reflect individual values, they may also relate to limited understanding of long‐term risks and benefits. Enhancing patients' understanding of treatment goals and outcomes may help ensure that expressed preferences are better informed and that trade‐offs align more closely with both personal values and clinical consequences [30].

In addition, our mWTP analysis further highlighted a pronounced discrepancy in how patients and physicians view treatment costs. For several medication attributes, physicians believed patients should be willing to pay substantially more than they actually were; for example, the physician mWTP for cardiovascular benefit was approximately three‐fold higher than the corresponding patient estimate. Similar patterns have been reported in other disease areas, where physicians systematically overestimate patients' willingness to pay for therapeutic gains or risk reductions [31, 32, 33]. Together, these findings suggest that physicians may underestimate the extent to which affordability constrains patients' treatment choices. As with other DCE‐based WTP estimates, our mWTP values are inherently context‐specific and shaped by the prevailing insurance and cost‐sharing arrangements; however, they primarily capture respondents' marginal trade‐offs between efficacy, safety and cost, so the relative pattern of WTP across attributes is likely to be more informative than the absolute monetary amounts when considering other settings [34, 35, 36]. In China, affordability constraints are likely driven by heterogeneity in reimbursement rates and co‐payment rules across insurance schemes and regions, as well as variation in outpatient chronic disease coverage and local benefit design. Such institutional differences can translate the same listed medicine into markedly different monthly out‐of‐pocket payments for patients, thereby shaping the trade‐offs observed in stated preferences [37, 38]. In health systems where out‐of‐pocket payments still account for a substantial share of diabetes‐related expenditures, including the Chinese setting of our study, these findings underline the importance of treating affordability as a core dimension of value—alongside clinical benefit and safety—when designing benefit packages and structuring shared decision‐making, so as to support long‐term adherence and glycemic control [22, 26, 27, 39].

Furthermore, latent class analysis found that certain preference types existed among patients but had no corresponding class among physicians. For example, Class 4 patients, the largest group (31%), were highly sensitive to out‐of‐pocket costs, with limited concern for treatment efficacy or side effects. This cost‐driven preference type was not observed among physician classes, highlighting the preference gap in value prioritization between patients and physicians. These findings suggest that physicians cannot assume that their own value hierarchy mirrors that of their patients; instead, they should actively elicit individual preferences—particularly around treatment costs—and incorporate them into treatment planning. For strongly cost‐sensitive patients, there is also a risk that financial considerations overshadow awareness of long‐term cardiovascular benefits and hypoglycemia risks; targeted risk communication and simple decision aids may help rebalance these trade‐offs without imposing unaffordable regimens, thereby supporting adherence and patient satisfaction [33, 40]. For example, a standardized one‐page decision template could summarize (i) expected HbA1c reduction, (ii) absolute hypoglycemia risk over the first 6 months, (iii) key adverse events, (iv) cardiovascular benefit evidence (yes/no), and (v) estimated monthly out‐of‐pocket cost under typical reimbursement scenarios, together with a brief values‐clarification prompt and short educational explanations to help patients understand why these attributes matter for both short‐term symptom control and longer‐term outcomes [41, 42].

This study also has limitations regarding the analysis of preference differences between patients and physicians. First, although the physician sample size was considerably smaller than that of the patient group, this reflects, to some extent, the naturally smaller population of physicians involved in diabetes care relative to the patient population. Nevertheless, the limited number of physician responses may still affect the precision of preference estimates and the robustness of comparative analyses. Second, to avoid respondent fatigue and reduce task complexity in the DCE, nausea, vomiting, and diarrhea were combined into a single attribute—“gastrointestinal adverse events.” This approach did not account for the individual weight or severity of each symptom, potentially obscuring finer preference distinctions. Third, treatment efficacy was presented as HbA1c reduction using both qualitative and quantitative descriptions, and some patients may still have had difficulty understanding this clinical indicator. Future studies could consider more intuitive benefit attributes (e.g., changes in life expectancy or risk of complications). In this study, we tried to mitigate comprehension issues by conducting preliminary qualitative work, expert consultations and 30 rounds of cognitive pretesting to improve the clarity of the survey instrument and the interpretability of the results. Fourth, the survey design did not include an opt‐out option, which may have introduced some degree of forced‐choice bias, although this reflects the clinical reality of T2D treatment where pharmacological intervention is typically necessary. Fifth, mWTP estimates are inherently context‐specific and influenced by health system structure and financing arrangements. Therefore, absolute WTP values from the Chinese setting should not be directly extrapolated to substantially different systems. However, the study adhered to ISPOR good practice recommendations, employing rigorous sampling and appropriate econometric modeling to minimize bias and strengthen internal validity [25, 43]. Although transferability of point estimates may be limited, the relative preference patterns regarding efficacy, safety, and cost may offer policy‐relevant insights for other health care settings. Additionally, the DCE design is inherently based on hypothetical scenarios. Validation studies and a recent meta‐analysis that compared DCE‐based predictions with observed treatment and prescribing‐related behaviors generally report a moderate level of concordance at the group level, suggesting that DCEs can provide useful, albeit indirect, evidence on real‐world decision‐making [40, 41]. In our study, the results should therefore be interpreted primarily as differences in stated preferences between patients and physicians, while recognizing that such differences are likely to be informative for clinical practice; future research should explicitly link DCE‐derived preferences with prescribing or insurance claims data to further assess external validity. Sixth, the use of different survey modes—face‐to‐face interviews for patients and online questionnaires for physicians—may have introduced some mode‐related bias [44]. In our study, this trade‐off was partly intentional: face‐to‐face interviews were used for patients to ensure adequate comprehension and data completeness, whereas self‐administered online questionnaires were adopted for physicians to accommodate their busy schedules and facilitate participation. Although using different modes implies that a small portion of the observed patient–physician differences may reflect mode effects rather than pure preference heterogeneity, several design features helped mitigate this concern, including an identical experimental design, harmonized attribute descriptions, and standardized instructions across modes.

## 5 Conclusion

In conclusion, this study identified differences in preferences between patients and physicians when selecting anti‐hyperglycemic medication. Patients prioritize monthly out‐of‐pocket cost and treatment efficacy, whereas physicians emphasize hypoglycemic risk and cardiovascular benefits. Out‐of‐pocket cost and gastrointestinal adverse events are common concerns for both groups. Physicians should recognize that their own preferences may not align with those of their patients. A better understanding of patient preferences can enhance communication, promote individualized treatment plans, and improve the quality of shared decision‐making.

## Funding

This work was funded by the Department of Management Science, National Natural Science Foundation of China (grant no. 72074047).

## Disclosure

The authors have nothing to report.

## Conflicts of Interest

The authors declare no conflicts of interest.

## Supporting information


**Table S1:** Demographic characteristics: Physicians.
**Table S2:** Demographic characteristics: Patients.
**Table S3:** Model fit information for competing latent class model.
**Table S4:** Latent class model for preferences heterogeneity: Physicians.
**Table S5:** Latent class model for preferences heterogeneity: Patients.
**Figure S1:** Example of discrete choice experiment question (English version).
**Figure S2:** Example of discrete choice experiment question (Chinese version).

## References

[1] R. Rodriguez‐Gutierrez , M. R. Gionfriddo , N. S. Ospina , et al., “Shared Decision Making in Endocrinology: Present and Future Directions,” Lancet Diabetes & Endocrinology 4, no. 8 (2016): 706–716. 26915314 10.1016/S2213-8587(15)00468-4

[2] S. E. Inzucchi , R. M. Bergenstal , J. B. Buse , et al., “Management of Hyperglycemia in Type 2 Diabetes, 2015: A Patient‐Centered Approach: Update to a Position Statement of the American Diabetes Association and the European Association for the Study of Diabetes,” Diabetes Care 38, no. 1 (2015): 140–149. 25538310 10.2337/dc14-2441

[3] S. W. Lahiri , “Personalizing Type 2 Diabetes Management: Use of a Patient‐Centered Approach to Individualizing A1C Goals and Pharmacological Regimens,” Clinical Diabetes 35, no. 5 (2017): 321–328, 10.2337/cd17-0083. 29263575 PMC5734170

[4] A. C. Mühlbacher , A. Sadler , and C. Juhnke , “Personalized Diabetes Management: What Do Patients With Diabetes Mellitus Prefer? A Discrete Choice Experiment,” European Journal of Health Economics 22, no. 3 (2021): 425–443, 10.1007/s10198-021-01264-6. PMC7954752 33587221

[5] H. L. Gelhorn , S. M. Stringer , A. Brooks , et al., “Preferences for Medication Attributes Among Patients With Type 2 Diabetes Mellitus in the UK,” Diabetes, Obesity & Metabolism 15, no. 9 (2013): 802–809, 10.1111/dom.12091. 23464623

[6] A. Mühlbacher and S. Bethge , “What Matters in Type 2 Diabetes Mellitus Oral Treatment? A Discrete Choice Experiment to Evaluate Patient Preferences,” European Journal of Health Economics 17, no. 9 (2016): 1125–1140, 10.1007/s10198-015-0750-5. 26682548

[7] K. Doyle‐Delgado , J. J. Chamberlain , J. H. Shubrook , N. Skolnik , and J. Trujillo , “Pharmacologic Approaches to Glycemic Treatment of Type 2 Diabetes: Synopsis of the 2020 American Diabetes Association's Standards of Medical Care in Diabetes Clinical Guideline,” Annals of Internal Medicine 173, no. 10 (2020): 813–821. 32866414 10.7326/M20-2470

[8] R. C. Turner , C. A. Cull , V. Frighi , R. R. Holman , Group UPDS , and Group UPDS , “Glycemic Control With Diet, Sulfonylurea, Metformin, or Insulin in Patients With Type 2 Diabetes Mellitus: Progressive Requirement for Multiple Therapies (UKPDS 49),” JAMA 281, no. 21 (1999): 2005–2012. 10359389 10.1001/jama.281.21.2005

[9] N. Genere and V. M. Montori , “Newer Second‐Line Drugs for Diabetes Are Not More Cost‐Effective Than Sulfonylureas,” Annals of Internal Medicine 168, no. 2 (2018): JC8. 29335720 10.7326/ACPJC-2018-168-2-008

[10] S. E. Choi , S. A. Berkowitz , J. S. Yudkin , H. Naci , and S. Basu , “Personalizing Second‐Line Type 2 Diabetes Treatment Selection: Combining Network Meta‐Analysis, Individualized Risk, and Patient Preferences for Unified Decision Support,” Medical Decision Making 39, no. 3 (2019): 239–252. 30767632 10.1177/0272989X19829735 PMC6469997

[11] L. L. Humphrey , D. Kansagara , A. Qaseem , and Physicians* HVCCotACo , “World Health Organization Guidelines on Medicines for Diabetes Treatment Intensification: Commentary From the American College of Physicians High Value Care Committee,” American College of Physicians 169 (2018): 398–400. 10.7326/M18-1148 30178054

[12] S. Ozdemir , D. Baid , N. R. Verghese , et al., “Patient Preferences for Medications in Managing Type 2 Diabetes Mellitus: A Discrete Choice Experiment,” Value in Health 23, no. 7 (2020): 842–850, 10.1016/j.jval.2020.01.023. 32762985

[13] T. T. Chen , K. P. Chung , H. C. Huang , L. N. Man , and M. S. Lai , “Using Discrete Choice Experiment to Elicit Doctors' Preferences for the Report Card Design of Diabetes Care in Taiwan—A Pilot Study,” Journal of Evaluation in Clinical Practice 16, no. 1 (2010): 14–20, 10.1111/j.1365-2753.2008.01105.x. 20367811

[14] C. Carmona , J. Crutwell , M. Burnham , and L. Polak , “Shared Decision‐Making: Summary of NICE Guidance,” BMJ 373 (2021): n1430. 34140279 10.1136/bmj.n1430

[15] M. Zhang , X. He , J. Wu , and F. Xie , “Differences Between Physician and Patient Preferences for Cancer Treatments: A Systematic Review,” BMC Cancer 23, no. 1 (2023): 1126. 37980466 10.1186/s12885-023-11598-4 PMC10657542

[16] N. Umar , D. Litaker , M.‐L. Schaarschmidt , W. K. Peitsch , A. Schmieder , and D. D. Terris , “Outcomes Associated With Matching Patients' Treatment Preferences to Physicians' Recommendations: Study Methodology,” BMC Health Services Research 12, no. 1 (2012): 1, 10.1186/1472-6963-12-1. 22214259 PMC3276415

[17] R. E. Say and R. Thomson , “The Importance of Patient Preferences in Treatment Decisions—Challenges for Doctors,” BMJ 327, no. 7414 (2003): 542–545. 12958116 10.1136/bmj.327.7414.542 PMC192849

[18] G. Forsander , S. Stallknecht , U. Samuelsson , C. Marcus , and M. Bøgelund , “Preferences for Treatment Among Adolescents With Type 1 Diabetes: A National Study Using a Discrete Choice Experiment Model,” Diabetic Medicine 35, no. 5 (2018): 621–629, 10.1111/dme.13592. 29381816

[19] A. B. Hauber , S. Han , J. C. Yang , et al., “Effect of Pill Burden on Dosing Preferences, Willingness to Pay, and Likely Adherence Among Patients With Type 2 Diabetes,” Patient Preference and Adherence 7 (2013): 937–949, 10.2147/ppa.S43465. 24086104 PMC3786815

[20] A. F. Mohamed , J. Zhang , F. R. Johnson , et al., “Avoidance of Weight Gain Is Important for Oral Type 2 Diabetes Treatments in Sweden and Germany: Patient Preferences,” Diabetes & Metabolism 39, no. 5 (2013): 397–403, 10.1016/j.diabet.2013.06.001. 23880594

[21] S. Liu , J. Liu , L. Si , et al., “Patient Preferences for Anti‐Hyperglycaemic Medication for Type 2 Diabetes Mellitus in China: Findings From a National Survey,” BMJ Global Health 8, no. 4 (2023): e010942. 10.1136/bmjgh-2022-010942 PMC10106002 37041021

[22] Y. Lv , R. Ren , C. Tang , K. Song , S. Li , and H. Wang , “Preferences for Patients With Type 2 Diabetes Mellitus for Medications in Shandong Province, China: A Discrete Choice Experiment,” Patient Preference and Adherence 16 (2022): 2335–2344, 10.2147/ppa.S367985. 36046499 PMC9423121

[23] L. B. von Arx , F. R. Johnson , M. R. Mørkbak , and T. Kjær , “Be Careful What You Ask for: Effects of Benefit Descriptions on Diabetes Patients' Benefit‐Risk Tradeoff Preferences,” Value in Health 20, no. 4 (2017): 670–678, 10.1016/j.jval.2016.11.023. 28408010

[24] O. Maslovskaya , B. Struminskaya , and G. Durrant , The Future of Online Data Collection in Social Surveys: Challenges, Developments and Applications (Oxford University Press, 2022), 768–772.

[25] A. B. Hauber , J. M. González , C. G. Groothuis‐Oudshoorn , et al., “Statistical Methods for the Analysis of Discrete Choice Experiments: A Report of the ISPOR Conjoint Analysis Good Research Practices Task Force,” Value in Health 19, no. 4 (2016): 300–315, 10.1016/j.jval.2016.04.004. 27325321

[26] Y. Huang , Q. Huang , A. Xu , M. Lu , and X. Xi , “Patient Preferences for Diabetes Treatment Among People With Type 2 Diabetes Mellitus in China: A Discrete Choice Experiment,” Frontiers in Public Health 9 (2021): 782964, 10.3389/fpubh.2021.782964. 35178370 PMC8846300

[27] L. Zheng , S. Liu , Z. Liu , et al., “Eliciting Medication Preferences of Patients With Type 2 Diabetes Under Different Insurance Coverages in China,” Frontiers in Public Health 12 (2024): 1413642, 10.3389/fpubh.2024.1413642. 39525467 PMC11544538

[28] A. C. Mühlbacher and C. Juhnke , “Patient Preferences Versus Physicians' Judgement: Does It Make a Difference in Healthcare Decision Making?,” Applied Health Economics and Health Policy 11 (2013): 163–180. 23529716 10.1007/s40258-013-0023-3

[29] M. A. Powers , J. K. Bardsley , M. Cypress , et al., “Diabetes Self‐Management Education and Support in Adults With Type 2 Diabetes: A Consensus Report of the American Diabetes Association, the Association of Diabetes Care & Education Specialists, the Academy of Nutrition and Dietetics, the American Academy of Family Physicians, the American Academy of PAs, the American Association of Nurse Practitioners, and the American Pharmacists Association,” Diabetes Educator 46, no. 4 (2020): 350–369, 10.1177/0145721720930959. 32510275

[30] M. Peimani , A. L. Stewart , G. Garmaroudi , and E. Nasli‐Esfahani , “Shared Decision‐Making in Type 2 Diabetes: A Systematic Review of Patients' Preferences and Healthcare Providers' Perspectives,” BMC Health Services Research 25, no. 1 (2025): 39, 10.1186/s12913-024-12160-z. 39773273 PMC11705876

[31] G. M. Allan , J. Lexchin , and N. Wiebe , “Physician Awareness of Drug Cost: A Systematic Review,” PLoS Medicine 4, no. 9 (2007): e283, 10.1371/journal.pmed.0040283. 17896856 PMC1989748

[32] T. Schutte , J. Tichelaar , P. Nanayakkara , M. Richir , and M. van Agtmael , “Students and Doctors Are Unaware of the Cost of Drugs They Frequently Prescribe,” Basic & Clinical Pharmacology & Toxicology 120, no. 3 (2017): 278–283, 10.1111/bcpt.12678. 27639184

[33] C. E. Sloan , L. Millo , S. Gutterman , and P. A. Ubel , “Accuracy of Physician Estimates of Out‐Of‐Pocket Costs for Medication Filling,” JAMA Network Open 4, no. 11 (2021): e2133188, 10.1001/jamanetworkopen.2021.33188. 34739059 PMC8571653

[34] E. W. de Bekker‐Grob , M. Ryan , and K. Gerard , “Discrete Choice Experiments in Health Economics: A Review of the Literature,” Health Economics 21, no. 2 (2012): 145–172, 10.1002/hec.1697. 22223558

[35] V. Soekhai , E. W. de Bekker‐Grob , A. R. Ellis , and C. M. Vass , “Discrete Choice Experiments in Health Economics: Past, Present and Future,” Pharmaco Economics 37, no. 2 (2019): 201–226, 10.1007/s40273-018-0734-2. PMC6386055 30392040

[36] J. Zhao , H. Wang , X. Li , et al., “Importance of Attributes and Willingness to Pay for Oral Anticoagulant Therapy in Patients With Atrial Fibrillation in China: A Discrete Choice Experiment,” PLoS Medicine 18, no. 8 (2021): e1003730, 10.1371/journal.pmed.1003730. 34437553 PMC8432810

[37] Y. Ren , Z. Zhou , D. Cao , et al., “Did the Integrated Urban and Rural Resident Basic Medical Insurance Improve Benefit Equity in China?,” Value in Health 25, no. 9 (2022): 1548–1558, 10.1016/j.jval.2022.03.007. 35514010

[38] T. Zhang and M. Chen , “Long‐Term Effects of Establishing Outpatient Pooling Funds on Financial Protection: Empirical Evidence From a Quasi‐Natural Experiment in China,” International Journal of Health Planning and Management 40, no. 1 (2025): 204–223, 10.1002/hpm.3859. 39385320

[39] J. Geng , H. Bao , Z. Feng , J. Meng , X. Yu , and H. Yu , “Investigating Patients' Preferences for New Anti‐Diabetic Drugs to Inform Public Health Insurance Coverage Decisions: A Discrete Choice Experiment in China,” BMC Public Health 22, no. 1 (2022): 1860. 36199056 10.1186/s12889-022-14244-z PMC9533494

[40] C. Brown and M. Venetis , “Communicative Pathways Predicting Adherence in Type II Diabetic Patients: A Mediation Analysis,” Health Communication 38 (2022): 3051–3068, 10.1080/10410236.2022.2131980. 36259091

[41] W. Du , P. Liu , and W. Xu , “Effects of Decreasing the Out‐Of‐Pocket Expenses for Outpatient Care on Health‐Seeking Behaviors, Health Outcomes and Medical Expenses of People With Diabetes: Evidence From China,” International Journal for Equity in Health 21, no. 1 (2022): 162, 10.1186/s12939-022-01775-5. 36384591 PMC9667616

[42] R. Richter , J. Jansen , I. Bongaerts , O. Damman , J. Rademakers , and T. van der Weijden , “Communication of Benefits and Harms in Shared Decision Making With Patients With Limited Health Literacy: A Systematic Review of Risk Communication Strategies,” Patient Education and Counseling 116 (2023): 107944, 10.1016/j.pec.2023.107944. 37619376

[43] F. Reed Johnson , E. Lancsar , D. Marshall , et al., “Constructing Experimental Designs for Discrete‐Choice Experiments: Report of the ISPOR Conjoint Analysis Experimental Design Good Research Practices Task Force,” Value in Health 16, no. 1 (2013): 3–13, 10.1016/j.jval.2012.08.2223. 23337210

[44] R. Jiang , E. Pullenayegum , J. W. Shaw , et al., “Comparison of Preferences and Data Quality Between Discrete Choice Experiments Conducted in Online and Face‐To‐Face Respondents,” Medical Decision Making 43, no. 6 (2023): 667–679. 37199407 10.1177/0272989X231171912 PMC10422849

