# Supplementary material for: Is There Any Difference in Preferences Between Patients and Physicians? Evidence From Anti‐Hyperglycemic Medications Choices for Type 2 Diabetes
Source: J Diabetes. 2026 Apr 15;18(4):e70220. doi: 10.1111/1753-0407.70220 (PMC13083033; doi:10.1111/1753-0407.70220)
Supplement: Supplementary file 1 — Table S1: Demographic characteristics: Physicians. Table S2: Demographic characteristics: Patients. Table S3: Model fit information for competing latent class model. Table S4: Latent class model for preferences heterogeneity: Physicians. Table S5: Latent class model for preferences heterogeneity: Patients. Figure S1: Example of discrete choice experiment question (English version). Figure S2: Example of discrete choice experiment question (Chinese version). [file JDB-18-e70220-s001.doc]

**Supplemental material**

Table S1. Demographic characteristics: physicians

| **Characteristics (N=186)** | **Category** | **Count** | **Percentage** |
| --- | --- | --- | --- |
| **Gender** | Male | 80 | 43.01% |
|  | Female | 106 | 56.99% |
| **Age** | 18–29 years | 10 | 5.38% |
|  | 30–39 years | 99 | 53.23% |
|  | 40–49 years | 48 | 25.81% |
|  | 50–59 years | 29 | 15.59% |
| **Professional Title** | Resident Physician | 36 | 19.35% |
|  | Attending Physician | 82 | 44.09% |
|  | Associate Chief Physician | 50 | 26.88% |
|  | Chief Physician | 18 | 9.68% |
| **Duration of Work in Diabetes Care** | Less than 1 year | 8 | 4.30% |
|  | 1-3 years | 30 | 16.13% |
|  | 4-6 years | 38 | 20.43% |
|  | 7-10 years | 37 | 19.89% |
|  | More than 10 years | 73 | 39.25% |
| **Type of Healthcare Institution** | General Hospital | 98 | 52.69% |
|  | Specialized Hospital | 22 | 11.83% |
|  | Traditional Chinese Medicine Hospital | 10 | 5.38% |
|  | Community Health Center | 39 | 20.97% |
|  | Township Health Center | 4 | 2.15% |
|  | Village Clinic | 11 | 5.91% |
|  | Others | 2 | 1.08% |
| **Hospital Grade** | Tertiary Grade A | 71 | 54.62% |
|  | Tertiary Grade B | 17 | 13.08% |
|  | Secondary Grade A | 34 | 26.15% |
|  | Secondary Grade B | 1 | 0.77% |
|  | Others | 7 | 5.38% |

Table S2. Demographic characteristics: patients

| **Characteristics (N=1784)** | **Category** | **Count** | **Percentage** |
| --- | --- | --- | --- |
| **Gender** | Male | 908 | 50.90% |
|  | Female | 876 | 49.10% |
| **Age** | 18–29 years | 15 | 0.84% |
|  | 30–39 years | 91 | 5.10% |
|  | 40–49 years | 201 | 11.27% |
|  | 50–59 years | 503 | 28.20% |
|  | More than 60 years | 974 | 54.60% |
| **Height（cm）** |  | 164.4±8.07 |  |
| **Weight（kg）** |  | 64.2±11.2 |  |
| **BMI** |  | 23.8±4.8 |  |
| **Inpatient Status** | Yes | 520 | 29.15% |
|  | No | 1264 | 70.85% |
| **Place of Residence** | Urban | 1103 | 61.83% |
|  | Rural | 681 | 38.17% |
| **Marital Status** | Married | 1632 | 91.48% |
|  | Unmarried | 30 | 1.68% |
|  | Divorced | 18 | 1.01% |
|  | Widowed | 104 | 5.83% |
| **Education Level** | Elementary School or Below | 442 | 24.78% |
|  | Junior High School | 570 | 31.95% |
|  | Senior High School | 359 | 20.12% |
|  | Technical School | 106 | 5.94% |
|  | College | 151 | 8.46% |
|  | Bachelor’s Degree or Above | 156 | 8.74% |
| **Medical Insurance Type** | Urban Employee Medical Insurance | 796 | 44.62% |
|  | Urban-Rural Resident (New Rural Cooperative) Insurance | 963 | 53.98% |
|  | Commercial Medical Insurance | 17 | 0.95% |
|  | Uninsured | 19 | 1.07% |
| **Annual Household Income** | Less than ¥20,000 | 414 | 23.21% |
|  | ¥20,000–¥40,000 | 580 | 32.51% |
|  | ¥50,000–¥70,000 | 315 | 17.66% |
|  | ¥80,000–¥100,000 | 368 | 20.63% |
|  | ¥110,000–¥130,000 | 47 | 2.63% |
|  | ¥140,000–¥160,000 | 24 | 1.35% |
|  | ¥170,000–¥200,000 | 14 | 0.78% |
|  | More than ¥200,000 | 22 | 1.23% |
| **Duration Since Diagnosis** | Less than 3 years | 296 | 16.59% |
|  | 3-5 years | 361 | 20.24% |
|  | 5-10 years | 476 | 26.68% |
|  | More than 10 years | 651 | 36.49% |
| **Fasting blood glucose** | More than 7.0 | 1145 | 64.18% |
|  | 6.1-7.0 | 420 | 23.54% |
|  | Less than 6.1 | 81 | 4.54% |
|  | Not clear | 138 | 7.74% |
| **Monthly out-of-pocket cost for anti-hyperglycemic medications** | ¥0-¥50 | 165 | 9.25% |
|  | ¥50-¥100 | 311 | 17.43% |
|  | ¥100-¥200 | 364 | 20.40% |
|  | ¥200-¥400 | 470 | 26.35% |
|  | ¥400-¥600 | 242 | 13.57% |
|  | More than ¥600 | 121 | 6.78% |
|  | Not taking medication | 111 | 6.22% |

Table S3. Model fit information for competing latent class model

|  | **Classes** | **LLF** | **df** | **BIC** | **AIC** |
| --- | --- | --- | --- | --- | --- |
| Physicians | 2 | -1206.82 | 33 | 2583.71 | 2479.65 |
|  | 3 | -1170.84 | 50 | 2599.34 | 2441.68 |
|  | 4 | -1155.56 | 67 | 2656.40 | 2445.13 |
|  | 5 | -1117.40 | 84 | 2667.67 | 2402.80 |
|  | 6 | -1074.48 | 101 | 2669.45 | 2350.97 |
| Patients | 2 | -5593.78 | 33 | 11434.62 | 11253.56 |
|  | 3 | -5446.28 | 50 | 11266.89 | 10992.56 |
|  | 4 | -5335.83 | 67 | 11173.27 | 10805.66 |
|  | 5 | -5262.17 | 84 | 11153.21 | 10692.33 |
|  | 6 | -5230.58 | 101 | 11217.31 | 10663.16 |

Table S4. Latent class model for preferences heterogeneity: Physicians

| **Attributes** | **Levels** | **Class1** | **Class2** | **Class3** | **Class4** | **Class5** | **Class6** |
| --- | --- | --- | --- | --- | --- | --- | --- |
| **Treatment efficacy/reduction in HbA1c** | 0.5% (ref.) |  |  |  |  |  |  |
|  | 1% | -0.50 | 1.27 | 0.41 | 1.09 | 4.89 | -4.35 |
|  | 1.50% | 0.25 | -2.37 | 0.36 | 0.73 | -24.73 | -3.59 |
|  | 2.50% | 0.82 | 4.82 | 1.21 | 0.10 | 13.09 | -4.47 |
| **Hypoglycaemic risk** | 30% (ref.) |  |  |  |  |  |  |
|  | 15% | -1.57 | 1.52 | 2.91 | 0.81 | 13.44 | -0.74 |
|  | 5% | -0.80 | 0.99 | 2.56 | 0.85 | -34.06 | 4.13 |
|  | 0% | 0.16 | 1.07 | 2.94 | 1.16 | -12.38 | 0.32 |
| **Gastrointestinal adverse events** | 40% (ref.) |  |  |  |  |  |  |
|  | 20% | -0.10 | 9.68 | -0.14 | 0.19 | 17.76 | 0.38 |
|  | 10% | -0.53 | 7.14 | -0.29 | 1.18 | -12.48 | 6.49 |
|  | 0% | -0.13 | 9.92 | 0.13 | 1.00 | -3.22 | 4.65 |
| **Weight change** | No change (ref.) | | |  |  |  |  |
|  | −2 kg | 0.59 | -2.96 | 1.59 | -0.57 | 24.29 | 0.57 |
|  | +1.5 kg | -0.43 | -8.68 | 0.64 | -0.16 | 2.83 | -5.69 |
|  | +3 kg | -0.16 | -3.72 | 1.80 | -0.72 | 35.70 | -6.40 |
| **Cardiovascular benefits** | No (ref.) |  |  |  |  |  |  |
|  | Yes | -0.16 | 7.64 | 0.95 | 0.75 | 24.49 | 2.09 |
| **Mode of administration** | Injection (ref.) | | |  |  |  |  |
|  | Pill | 0.47 | 2.34 | 0.53 | 0.23 | -12.89 | 1.54 |
| **Out-of-pocket cost#** | 0 CNY (ref.) |  |  |  |  |  |  |
|  | 600 CNY  (84.2 USD) | -2.40 | -3.60 | 0.60 | -0.60 | -1.20 | -0.60 |
| **Proportion** |  | 13% | 21% | 15% | 27% | 11% | 13% |

# Based on a currency exchange rate of 7.1217 CNY to 1.00 USD in 2024.

Table S5. Latent class model for preferences heterogeneity: Patients

| **Attributes** | **Levels** | **Class1** | **Class2** | **Class3** | **Class4** | **Class5** | **Class6** |
| --- | --- | --- | --- | --- | --- | --- | --- |
| **Treatment efficacy/reduction in HbA1c** | 0.5% (ref.) | | |  |  |  |  |
|  | 1% | 0.52 | 1.02 | 0.01 | 0.51 | 11.36 | 1.84 |
|  | 1.50% | 0.73 | 1.75 | 0.05 | 0.52 | 14.85 | 2.90 |
|  | 2.50% | 0.94 | 1.99 | -0.29 | 0.56 | 2.25 | 4.19 |
| **Hypoglycaemic risk** | 30% (ref.) | | |  |  |  |  |
|  | 15% | 1.04 | -0.19 | 0.69 | 0.36 | 1.59 | 0.47 |
|  | 5% | 1.89 | 0.80 | 0.78 | 0.20 | 25.13 | 0.20 |
|  | 0% | 2.08 | 0.51 | 1.16 | -0.16 | 2.48 | 0.36 |
| **Gastrointestinal adverse events** | 40% (ref.) | | |  |  |  |  |
|  | 20% | 0.95 | 0.83 | 0.04 | -0.56 | -39.26 | 0.90 |
|  | 10% | 1.15 | 0.06 | 0.47 | -0.48 | -24.67 | 1.17 |
|  | 0% | 2.44 | 1.46 | 0.45 | -0.03 | -31.23 | 1.01 |
| **Weight change** | No change (ref.) | | |  |  |  |  |
|  | −2 kg | 0.87 | -0.78 | 0.48 | -0.06 | 3.76 | -1.02 |
|  | +1.5 kg | 0.24 | -0.29 | 0.07 | -0.35 | 3.32 | -1.35 |
|  | +3 kg | 0.38 | -0.39 | 0.12 | -0.62 | -2.42 | -1.70 |
| **Cardiovascular benefits** | No (ref.) | | |  |  |  |  |
|  | Yes | -0.28 | 1.84 | 0.97 | 0.49 | -0.56 | 0.57 |
| **Mode of administration** | Injection (ref.) | | |  |  |  |  |
|  | Pill | 0.03 | 0.62 | -0.10 | 0.31 | 30.49 | 0.83 |
| **Out-of-pocket cost#** | 0 CNY (ref.) | | |  |  |  |  |
|  | 600 CNY  (84.2 USD) | -0.60 | -2.40 | 0.00 | -7.80 | 9.60 | -1.80 |
| **Proportion** |  | 15.7% | 16.3% | 12.9% | 30.8% | 10.6% | 13.7% |

# Based on a currency exchange rate of 7.1217 CNY to 1.00 USD in 2024.


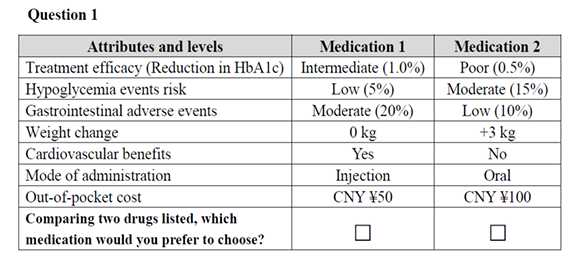


Figure S1. Example of discrete choice experiment question (English version)


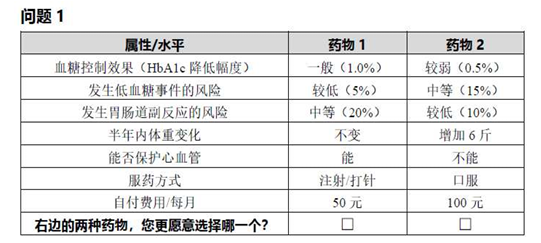


Figure S2 Example of discrete choice experiment question (Chinese version)
